# Production of Bio-Functional Protein through Revalorization of Apricot Kernel Cake

**DOI:** 10.3390/foods8080318

**Published:** 2019-08-06

**Authors:** Jelena Čakarević, Senka Vidović, Jelena Vladić, Aleksandra Gavarić, Stela Jokić, Nika Pavlović, Marijana Blažić, Ljiljana Popović

**Affiliations:** 1 Faculty of Technology, University of Novi Sad, Bul. cara Lazara 1, 21000 Novi Sad, Serbia; 2 Faculty of Food Technology Osijek, Josip Juraj Strossmayer University of Osijek, Franje Kuhača 20, 31000 Osijek, Croatia; 3 Faculty of Medicine, J.J. Strossmayer University of Osijek, Cara Hadrijana 10E, 31000 Osijek, Croatia; 4 Josip Juraj Strossmayer Square 9, Karlovac University of Applied Sciences, 47000 Karlovac, Croatia

**Keywords:** kernel protein, supercritical CO_2_ extraction, in vitro digestion, Angiotensin-I Converting Enzyme (ACE) inhibition, antioxidant activity

## Abstract

The study describes and compares bio-functional properties and thein vitrodigestibility of protein isolates from apricot oil cakes obtained by supercritical fluid extraction and cold pressing, as control. Protein isolates have the potential to be food ingredients with amygdalin contents in an amount considerably lower than regulatory. Isolates showed hypoglycemic activity, studied by the inhibition of *α*-glucosidase, also functional properties were determined.Good digestibility of proteins, which were done using gastrointestinal proteases (pepsin and pancreatin) were proven by Sodium Dodecyl Sulfate Polyacrylamide Gel Electrophoresis SDS-PAGE analysis. Moreover, it was evident that the protein isolates were completely digested. The biologically active potential of the digests was evaluated measuring in vitro antioxidant capacity by three complementary methods and enzyme inhibitory effects towards Angiotensin-I Converting Enzyme (ACE) related with the onset of hypertension. All hydrolysates act as a DPPH and ABTS scavenger, as a reducing agents and an ACE enzyme inhibitor. In conclusion, protein isolates obtained from apricot kernel cake showed to be a promising source of natural products for food applications, with good functional and bioactive properties and easy digestibility.

## 1. Introduction

The global production of apricot (*Prunusarmeniaca* L.) continually increases with demands from the fruit and food industry. In 2016 world apricot production amounted to 3.8 million tonnes [1]. Apricot kernel is a major by-product of the apricot fruit industry. There are huge economic and environmental benefits of the utilization of kernels from *Prunus* species (apricot, peach, plum, etc.). Generally, fruit kernels are valuable sources of oils that are rich in functional bioactive compounds such as essential fatty acids, tocochromanols, carotenoids, phytosterols and squalene. Several methods have been utilized worldwide to extract the oil from fruit kernels such as screw pressing, direct solvent extraction or pre-pressing followed by solvent extraction. These conventional methods have got a number of disadvantages: environmental pollution, high energy consumption, time-consuming, cost, requirement of organic solvent, and intensive separation technology.

With increasing interest in the “green chemistry” concept, different novel extraction techniques have been developed for extraction of bioactive compounds: microwave, ultrasonic or high pressure-assisted extraction supercritical fluid extraction (SFE) [2]. The goals of these techniques are reflected in increase of yield, reduction of time, solvent, energy consumption and environmental pollution.

SFE has been widely used since it enables the recovery of valuable food ingredients from natural matrices with high yield and the production of better quality products with improved functional and/or nutritional characteristics by operating under a wide range of conditions [3]. CO_2_ is the most preferred supercritical solvent due to a number of its advantages in food extractions, such as low critical temperature (31 °C), no toxicity, non-explosive nature, and low price [2].

However, oil extraction by supercritical CO_2_ also generated by-products (oil cakes/meals) where some of them could have a high protein content that have not been reused and characterized. Therefore, apricot kernel oil cake could constitute cheap source of proteins. Moreover, this is supported by the fact that essential amino acids in apricot kernel constituted 32–34% of the total amino acids, and major ones are arginine and leucine with 21.7–30.5 and 16.2–21.6 mmoL/100g meal, respectively [4].

Many authors have evaluated the potential of fruit seeds for production of bioactive peptides from isolated proteins [5]. Generally, release of bioactive peptides can be carried out by: in vitro enzymatic hydrolysis, gastrointestinal digestion and fermentation from the inactive parent proteins. These peptides can possess a wide variety of physiological functions including: antithrombotic, antimicrobial, cholesterol-lowering, antioxidative and ACE inhibitory/antihypertensive activities [5].

There has been a constant search for unconventional new protein sources for use as functional food ingredients and at the same time for nutritional supplements. To our knowledge, there has not been study on valorisation of by-products obtained after SFE into valuable protein product with potential bioactive and functional properties. Hence, this study proposes to evaluate the potential of apricot kernel oil cake for production of protein isolate whit good bio-functional properties, such as solubility, water and oil adsorption capacity, foaming and emulsifying ability and potential hypoglycaemic activity. Moreover, digestibility and ability for realising of bioactive hydrolysates with antioxidant and ACE inhibitory activity will be assayed.

## 2. Materials and Methods

### 2.1. Materials

The apricot kernels were obtained from Association of fruit brandy producers (Kneževivinogradi, Croatia). Oil cakes obtained by cold pressed (CP) and supercritical CO_2_ extraction (SFE) (Pavlović et al. 2018). Angiotensin-I Converting Enzyme (ACE) from rabbit lung, N-Hippuryl-His-Leu (HHL) hydrate, 2,2′-azino-bis (3-ethylbenzothiazoline-6-sulphonic acid) (ABTS), 2,2-diphenyl-1-pikryl-hydrazyl (DPPH) and pancreatin from porcine pancreas, with declared activity of 4 × USP specifications of units (0.03 N-benzoyl-L-tyrosine ethyl ester, BTEE (N-benzoyl-L-tyrosine ethyl ester) units/mg), Amigdalin (≥99%) standard, α-glucosidase from Saccharomyces cerevisiae with declared activity of 107 U/mg, 4-Nitrophenyl α-D- glucopyranoside were obtained from Sigma (St Louis, MO, USA). Pepsin from porcine stomach was purchased from AppliChem (Darmstadt, Germany), with declared activity of min 0.7 FIP U/mg. and min 1500 U/mg, respectively. All the other chemicals used for the experiments were of analytical or better grade. Proximate analysis was conducted using standard Association of Official Analytical Chemists (AOAC) (1990) methods.

### 2.2. Protein Extraction

Preparation of protein isolate from oil cakes obtained by cold pressing (PICP) and supercritical fluid extraction (PISFE) was performed by alkali extraction with isoelectric precipitation. The apricot cakes, which were defatted with hexane in ratio 1:5, were suspended in water solution at alkali pH 10.00 in ratio 1:10. After 30 min of extraction the slurry was filtered to remove the insoluble material. The filtrate that contain the dissolved proteins were precipitated by setting pH to 5.00 with 1 moL/dm^3^HCl. After centrifugation (Sorvall^®^ RC—5B Refrigerated Superpeed Centrifuge, Du Pont Instruments, Newtown, PA, USA at 10,000 rpm and 4 °C for 20 min, the precipitate was dried at 30 °C for 24 h. The dried precipitate was ground in coffee grinder to obtain PI powder. The yield of PI was 1.6 g/10 g oil cake.

### 2.3. Functional Properties of Protein Isolate

#### 2.3.1. Protein Solubility

The solubility of protein isolate (PI) was determined at pH range 2.0–9.0 and ionic strength range 0.1–1.0 moL/L NaCl. 10 mg of PI was weighted into Eppendorf tubes and added to 1 mL of a propriety buffer solution. The prepared samples were constantly stirred for 1 h at 25 °C, using a Thermo-Shaker TS-100C (BioSan, Latvia). Soluble proteins from supernatant were determined by the Lowry et al. [6] method.

#### 2.3.2. Water and Oil Absorption Capacity

Method described by Rodsamran and Sothornvit [7], with some modifications was used for determination of the water absorption capacity (WAC) and oil absorption capacity (OAC). Briefly, 100 mg of PI were mixed with 1 mL of distilled water or soybean oil in a vortex for 1 min. After incubation at 30 °C for 30 min in Thermo-Shaker TS-100C (BioSan, Latvia), the tubes were centrifuged at 14,500 rpm for 20 min (Eppendorf Mini-spin plus, Eppendorf AG., Hamburg, Germany) at room temperature. Water and oil were removed by inverting the tubes. The tubes with samples were weighed and WAC and OAC were expressed as g _(water or oil)_/g PI.

#### 2.3.3. Foaming Capacity and Stability

The foaming capacity (FC) and foaming stability (FS) were determined using a modified method described by Rodsamran and Sothornvit [7]. A half gram of PI was mixed with 50 mL buffer (0.1 moL/L sodium phosphate buffer pH 10.0) and then homogenized for 2 min in a high speed mixing in an Ultra-Turrax T25 at 5000 rpm for 2 min. The volume was recorded before and after whipping. The whipped protein solution was transferred into a 50 mL graduated cylinder and the volume was recorded at 0 min (V_0_) and 60 min (V_1_). The FC and FS were expressed, in percent, as the V_0_ and V_1_ divided by the total volume (× 100).

#### 2.3.4. Preparation and Characterization of Emulsions

Emulsions of PIs were prepared and characterized according to method described by Bučko et al. [8].Sunflower oil in water emulsions were prepared by dispersing 15% of sunflower oil in a continuous phase by means of Ultraturrax Te25 (Janke& Kunkel, Germany) homogenizer at 15,000 rpm for 10 min at 35 °C. Concentration of PI in the continuous phase was 10 mg/mL and pH was 10.0. Volume weighted mean diameter (d 4,3) of oil droplets in emulsions was determined by Mastersizer Micro Particle Analyzer (Malvern Instruments Ltd.,Worcestershire, UK). Buffer solutions which corresponded to the measured emulsion were used to collect background data. Emulsions were dosed so that obscuration stays between 10 and 20%. During measurements pump rotation speed was kept at 1500 rpm. The reported d 4,3 values are average value of at least three measurements. Stability of emulsions was evaluated by the creaming test. Immediately after preparation, emulsions were transferred into 10 mL sealed graduated glass cylinders, and next 14 days at room temperature their creaming behaviour were observed. During the time, emulsions separate in an emulsion cream (top) and an emulsion serum (bottom) layer. Changes in a creaming index, CI, were visually monitored, where the creaming index is:

CI (%) = V_S_/V_E l_ × 100

where Vsis volume of the serum layer and V_E_ is volume of the emulsion.

### 2.4. Determination of Amygdalin Content

Method was made according to Bolarinwaa et al. [9], with some corrections. Briefly, 2 g of protein cakes and PI were measured into a round-bottom flask (100 mL), and then added 50 mL ethanol and the mixture was boiled under reflux during 120 min at 78.5 °C. In the end of extraction, the extracts were filtered and evaporated under vacuum to remove ethanol. On this way samples were dissolved in the water andanalyzed by High-Performance Liquid Chromatography HPLC (Agilent 1290 Infinity I HPLC system with an Agilent DAD detector, Santa Clara, CA, USA. The column for separation was used a SupelcoAnalitycal HS-C18 column (4.6 × 250 mm, 5 µm) Sigma-Aldrich (St Louis, MO, USA). The chromatographic conditions were: flow rate 1 mL/min, temperature 20 °C, injection volume 20 µL and UV detection (Agilent DAD detector, Santa Clara, United States) at 210 nm. Mobil phase consisted of distillation water: methanol (75:25 *v*/*v*).

### 2.5. α-Glucosidase Inhibitory Potential

Assay for this method was described by Chan et al [10].

### 2.6. Protein Digestion

PIs were hydrolysed using gastrointestinal proteases in order to simulate the human gastrointestinal environment. Digestion was performed in a glass reactor using a combination of two enzymes, pepsin (E/S 1/25) and pancreatin (E/S 1/50), at 37 °C during 4 h. Briefly, the PI suspension in water (2.5 g/100 cm^3^) was pre-incubated at 37 °C, then adjusted to pH 2.5 and added pepsin. After 120 min gastric digestion, pH of the solution was adjusted to pH 7.00 and added pancreatin and intestinal digestion was carried out next 120 min. After 120 min the reaction mixture was heated at 100 °C for 5 min and centrifuged using Sorvall^®^RC—5B Refrigerated Super Speed Centrifuge (Du Pont Instruments, Newtown, PA, USA) at G-force 9220 and 4 °C for 10 min. The collected supernatants were further analysed.

### 2.7. Determination of the Degree of Hydrolysis

The degree of the hydrolysis (DH) was set by method described by Popović et al. [11].According to the method DH was expressed as the ratio of 0.44 moL/L TCA-soluble protein to total protein in the reaction mixture. Soluble proteins determined by the method of Lowry et al. (1951) using the bovine serum albumin as the standard protein.

### 2.8. SDS-PAGE

Proteins and hydrolysates were separated using sodium dodecyl sulfate polyacrylamide gel electrophoresis (SDS-PAGE), described by the method of Laemmli [12]. System used for separation consisted of a 4% (*w*/*v*) acrylamide stacking gel and a 10% (*w*/*v*) acrylamide separation gel.

### 2.9. Determination of Antioxidant Activity

#### 2.9.1. DPPH Radical Scavenging Assay

The assays were carried out according to the method by Morales and Jiménez-Pérez [13] with some modifications. DPPH radical scavenging activity was calculated according to the following equation:

AA (%) = (A_blank_-A_sample_)/A_blank_ × 100

where A_blank_ is the absorbance of the blank solution, A_sample_ is the absorbance of the sample solution.

#### 2.9.2. ABTS Radical Scavenging Activity Assay

The assay as described by [11], ABTS scavenging activity was calculated as:

Antioxidant activity (%) = (A_control_-A_sample_)/A_control_ × 100

where A_control_ is the concentration of ABTSin blank (in the presence of buffer instead of protein extract) and A_sample_ is the concentration of ABTSin the sample (in the presence of protein extract), after 10 min of reaction.

#### 2.9.3. Reducing Power

For determination of the reducing power it was used a method [11]. The sample solution of a protein hydrolysates (1 mL) was mixed with 2.5 mL of a phosphate buffer (0.2 moL/L, pH 6.6) and 2.5 mL of 1% potassium ferricyanide. The cuvettes with the mixture were incubated in water bath at 50 °C for 20 min. After incubation, in the samples was added 2.5 mL of 10% TCA and mixture wascentrifuged. The supernatant was diluted with water in the ratio 1:1 and then addition of 0.1% ferric chloride in the ratio 10:1. The mixture was incubated at 50 °C for 10 min, and after that the absorbance was measured at 700 nm (T80/T80+ UV-Vis Spectrophotometer, PG instruments Ltd., Leicestershire, UK. Increase of absorbance indicates increase of reducing power.

### 2.10. Assay of ACE-Inhibitory Activity

The ACE- inhibitory activity of the hydrolysates was measured following the method described by Yoshi-Stark, Bez, Wada&Wäsche [14]. Method is based on the reaction of hipuril-L-histidyl-leucine (HHL) into hippuric acid (HA) by the action of angiotensin converting enzyme (ACE) under defined conditions (80 min at 37 °C).After reaction is quantitated by product of the reaction on the HPLC equipment (1100 Agilent with an Agilent DAD detector, Agilent DAD detector, Santa Clara, CA, USA. The chromatographic conditions were: flow rate 1 mL/min, temperature 23 °C, injection volume 20 µL and UV detection at 228 nm. Mobil phase was 50% of methanol with 1% (*v*/*v*) TFA (trifluoroacetic acid) in double-distilled water. Inhibition of ACE was calculated by equation:

ACE inhibition (%) = (HA_blank_ − HA_sample_)/HA_blank_ × 100

where HA_blank_ is the peak area without ACE inhibition and HA_sample_ is the peak area in the sample.

### 2.11. Statistical Analysis

The data were in triplicate, and subjected to statistical analysis, using analysis of variance (ANOVA) to determine significant differences between the samples (*p* < 0.05). Differences between the treatment means were separated using Duncan’s multiple range tests.

## 3. Results

### 3.1. Proximate Composition and Amygdalin Content

The apricot stones generally contain a seed with a high content of proteins and lipids. According some authors apricot kernel contained 28% protein and 52% oil [15]. Proximate composition of apricot oil cakes, as well as content of protein in protein isolates are shown in Table 1. Protein was the major component in both cakes, followed by carbohydrates and crude fibres. Values obtained for proteins (about 42 g/100 g) were higher than those in the bibliography [5]. PIs, obtained from these cakes by isoelectric precipitation, have protein content 70.81 g/100 g for PISFE and 84.44 g/100 g for PICP.

Nevertheless, one limitation to the exploitation of this sustainable source for protein and oil production is the presence of cyanogenic glycosides such as amygdalin. The hydrolysis of amygdalin results in benzaldehyde, with a typical bitter taste, and cyanide. The European Food Safety Authority establishes a maximum level of cyanide of 50 mg/kg in nougat, marzipan or their substitutes or similar products, 5 mg/kg in canned stoned fruits, and 35 mg/kg in alcoholic beverages. Amygdaline was detected in PICP in amount considerably lower than those in the regulative, while in PISFE amygdaline did not detect (Table 1). Similar reported Garcia and co-workers [5]. This indicates that the extraction process affects the presence of amygdalin in the PI [9]. Hence, these protein isolates could be safely used in different protein food formulation or as source of bioactive peptides.

### 3.2. Functional Properties of Protein Isolate

**Figure 1:**
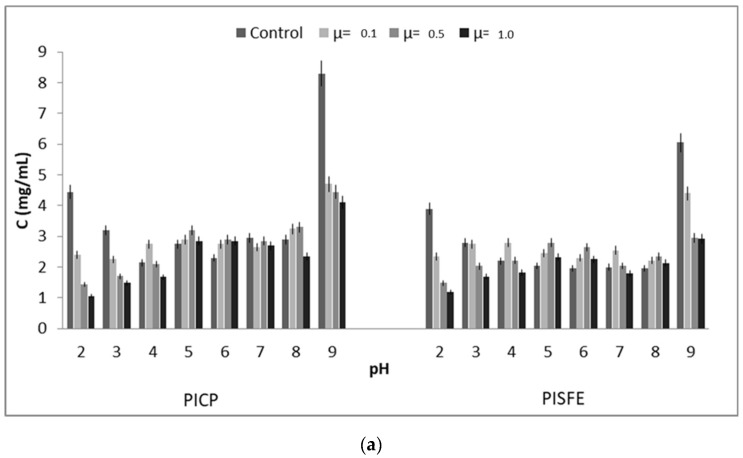

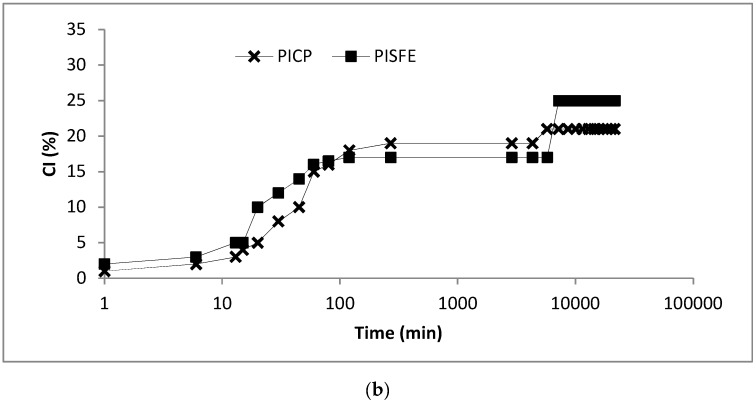
(**a**) Influence of pH and ionic strength (µ) on protein solubility of protein isolates of apricot oil cake protein isolates obtained by cold pressing (PICP) and supercritical fluid extraction (PISFE). Values represent the mean±standard deviation (SD) of *n* = 3 replicate analysis; (**b**) Creaming index of protein isolates obtained by cold pressing (CP) (×) and supercritical fluid extraction (SFE) (■) stabilized emulsions during 14 days of storage.

The functional properties influence the behaviour of proteins in food systems during processing, storage, preparation and consumption. Solubility is one of the most important characteristics of proteins because it directly affects the other functional properties. The protein solubility profiles of PICP and PISFE in different pH, and ionic strength are shown in Figure 1a. For both PIs, the lowest solubility of 2.2 mg/mL was achieved at pH 4.0, and maximal solubility was at pH 9.0 (6.1 mg/mL for SFE and 8.3 mg/mL for CP). Similar was obtained for solubility of apricot cake protein with values of 5 and 8.7 mg/mL at pH 4.0 and pH 8.0, respectively [16]. According to results obtained in this work, pI for extracted proteins is at pH = 4.0, what is in correlation with general trend that most of food proteins are acidic, with isoelectric point at pH 4.0–5.0 and with maximum solubility at alkaline pH [8]. Ionic strength has also influence on protein solubility, mostly at the pH extremes (pH 2.0 and pH 9.0) as salting-out effect. At pH values from pH = 4.0 to pH = 8.0 slightly salting-in effect takes place, with a minor increasing of solubility.

WAC and OAC influence the texture and mouthfeel characteristics of foods and food products. The WAC and OAC significantly differed among two PIs, their values were reported in Table 1. The present WAC and OAC values were lower than the reported value for apricot protein isolate [16].

EC was measured in this work as oil droplet size since smaller droplets reflect high emulsifying ability of a protein. The mean droplet diameter of the emulsions is presented in Table 1. The values for CPPI and SFEPI stabilised-emulsion were 3.29 ± 0.1 μm and 3.38 ± 0.4 µm, respectively, which are smaller or similar than the droplet size in some other PI stabilised-emulsions [8]. Creaming indexes of emulsions as a function of storage time are shown in Figure 1b. The emulsions, prepared in this research, were susceptible to creaming instability and they almost fully separated in serum and cream layer within 60 min after preparation.

Table 1, also, showed the FC and FS values for PIs. The FC and FS values obtained for both isolates were similar to each other and consistent with reports for other protein isolates [17]. These PIs could be considered to be a suitable foaming ingredient for use in different food products, such as cakes, bread, whipped cream, ice cream and some confectionery products.

### 3.3. A-Glucosidase Inhibitory Activity

**Figure 2:**
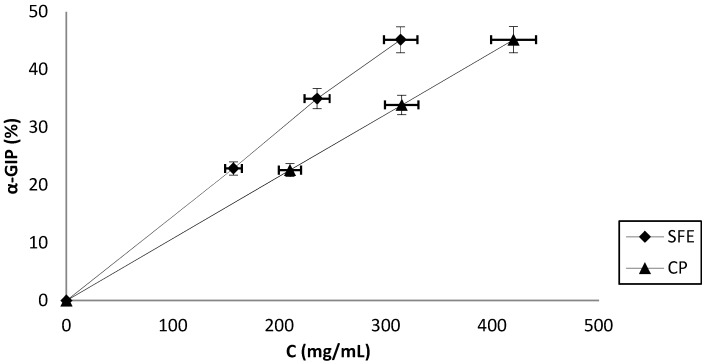
α-glucosidase inhibition potential (α-GIP) of apricot oil cake protein isolates obtained by cold pressing (CP) and supercritical fluid extraction (SFE) at different concentrations. Values represent the mean±standard deviation (SD) of *n* = 3 replicate analysis.

Diabetes mellitus characterized by hyperglycemia is important health issues worldwide. Diabetes mellitus is spreading very rapidly while currently available drugs, acarbose and miglitol, possess many gastrointestinal side effects on diabetics. α-Glucosidase inhibitors is an effective approach for controlling the postprandial and fasting blood glucose levels. Generally, there is a huge interest for efficacious α-glucosidase inhibitors with low side effects. Several plants and their extracts have been evaluated for their potential role against α-glucosidase enzyme. Besides phenolic components, proteins and their hydrolysates have also attracted attentions. The α-glucosidase inhibition of PICP and PISFE are shown in Figure 2, both isolates were capable of inhibiting the activity of α-glucosidase in a concentration-dependent manner. Slightly higher inhibition was observed in PISFE with the IC_50_ value of 343.58 mg/mL which was in a range with a reported IC_50_ values for some other protein compounds [18].

### 3.4. Digestibility of Protein Isolates

**Figure 3:**
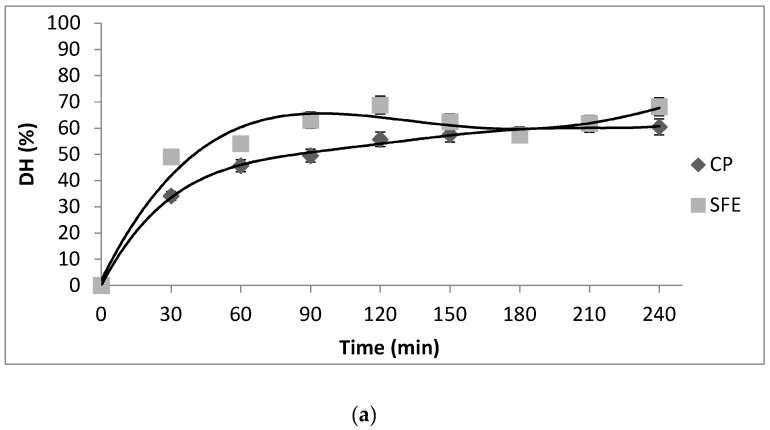

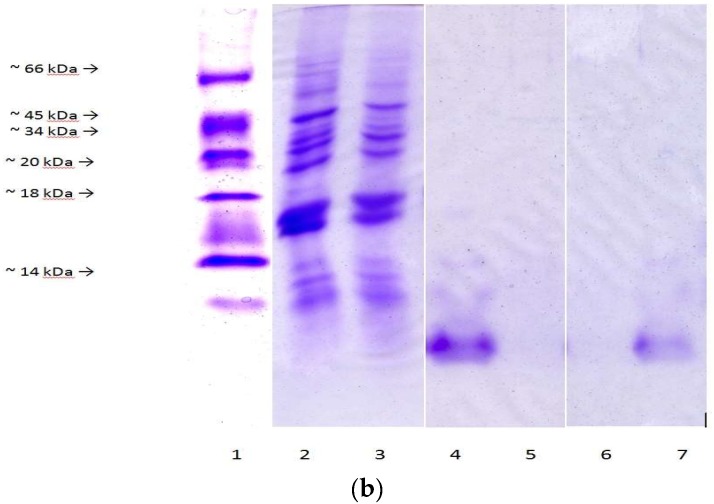
(**a**) Degree of hydrolysis (DH) of apricot oil cake protein isolates obtained by cold pressing (CP) and supercritical fluid extraction (SFE) during in vitro digestion first by pepsin 120 min and then with pancreatin during next 120 min. Values represent the mean±standard deviation (SD) of *n* = 3 replicate analysis; (**b**) SDS-PAGE analysis of apricot oil cake protein isolates obtained by cold pressing (CP) (line 2) and supercritical fluid extraction (SFE) (line 3) and their products of in vitro digestion.

DH was parameter for monitoring the protein hydrolysis. During simulated digestion, the hydrolysis rates of both investigated PIs followed a same trend, Figure 3a. After pepsin digestion, the DH of PICP and PISFE reached 55.71 ± 2.21% and 68.1 ± 1.82%, respectively.Further, subsequent digestion by pancreatin, not led to increase of DH in both PI digests, and final DH values were 57.3 ± 2.81% for CP and 68.2 ± 2.48% for SFE.

The protein profiles of native and digested PICP and PISFE were observed by SDS-PAGE, Figure 3b. Both native proteins showed the presence of three groups of intense bands, correspond to polypeptide complexes with molecular weight under 10 kDa, between 14–18 kDa and 20–45 kDa. Homogeneity in the profiles of major polypeptide bands indicates that total soluble proteins were not affected by the type, or by the temperature applied in treatments. Similar results are reported for other Prunus genus seed [19]. The protein patterns obtained after digestion with pepsin of both PI showed lower molecular weight fragments notably bellow 10 kDa. During the second enzymatic step these fragments were further digested by pancreatin and all the bands were vanished, suggesting complete degradation of both PIs. According to other authors similar it happens to proteins from almond kernel after in vitro digestion [20].

All of these, above mentioned, show that apricot cakes protein could be easily digested. According to the Codex Alimentarius Commission-2003 approach it is recommended for the assessment of potential allergenicity of novel proteins to include an in vitro pepsin digestion assay. The digestibility of novel proteins in simulated gastric fluid is considered to be an indicator of reduced risk of allergenic potential in food.

### 3.5. Antioxidant Capacity of Digested PIs

**Figure 4:**
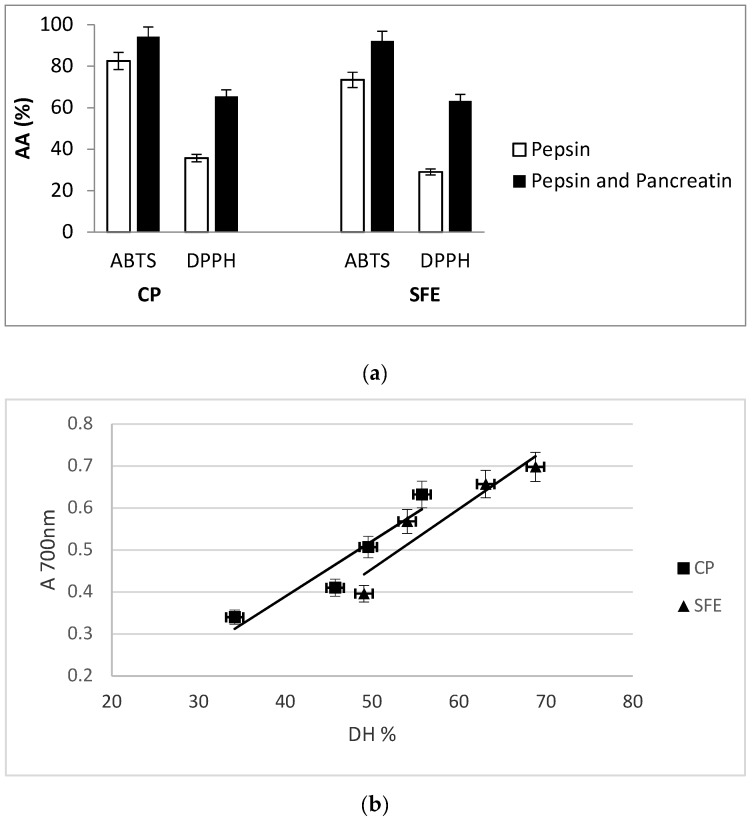
(**a)** Comparison of antioxidant activities (AA) of hydrolysates obtained after in vitro digestion of apricot oil cake protein isolates derived by cold pressing (CP) and supercritical fluid extraction (SFE) measured as 2,2′-azino-bis (3-ethylbenzothiazoline-6-sulphonic acid) (ABTS) and 2,2-diphenyl-1-pikryl-hydrazyl (DPPH) radical scavenging activity. Values represent the mean ± standard deviation (SD) of *n* = 3 replicate analysis; (**b**) Reducing power of hydrolysates obtained after in vitro digestion of apricot oil cake protein isolates derived by cold pressing (CP) and supercritical fluid extraction (SFE) with different degrees of hydrolyses (DH). Values represent the mean ± standard deviation (SD) of *n* = 3 replicate analysis.

The antioxidant capacity of a food or biological sample depends of many factors; hence it is advisable to use more than one assay for its determinations. Antioxidant capacity observed after both in vitro digestion steps for both PIs in every assay are presented in Figure 4a,b. In general, all digests showed radical scavenging capacity which values depend on the assay. After pepsin digestion, the DPPH radical scavenging activity of PICP and PISFE digests was 35.7% and 29.1%, respectively. After the next step of digestion, the scavenging activity increased to 65.2% and 63.2%, respectively (Figure 4a). Generally, during in vitro digestion both PIs were cleaved into small peptides and free amino acids which are reported to exert greater antioxidant activity than their parent proteins or large polypeptides. ABTS radical scavenging activity of both PIs was high immediately after incubation with pepsin and reached the values of 82.4% and 73.4%, for PICP and PISFE, respectively. After treatment with pancreatin, antioxidant activities continued rising, and in the final digests their values were 94.2% for PICP and 92.15% for PISFE (Figure 4a). Hydrolysates with different DH values, obtained during in vitro digestion, were tested on the reducing power, Figure 4b. There is reducing ability which increases with increasing of DH value. This is in accordance with the reports of other authors who suggested that more electron-dense amino acid side residue chain groups became exposed and an increased availability of free amino acids during digestion provided an additional source of protons and electrons to maintain a high redox potential with the increase in DH [21]. Hydrolysates obtained from PISFE showed slightly higher values. Based on the above results, it could be concluded that oil extraction treatments had no influence on ability of resultant digests to act as a radical quenchers and reducing agents.

### 3.6. ACE Inhibitory Capacity of Digested PIs

**Figure 5:**
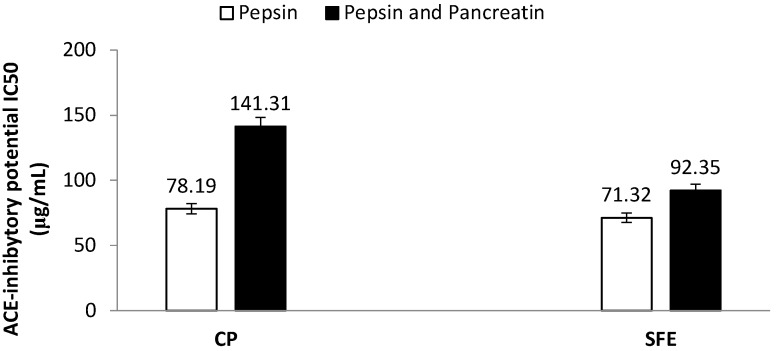
Comparison of Angiotensin-I Converting Enzyme (ACE) inhibitory potential of hydrolysates obtained after in vitro digestion of apricot oil cake protein isolates derived by cold pressing (CP) and supercritical fluid extraction (SFE). Values represent the mean ± standard deviation (SD) of *n* = 3 replicate analysis.

ACE inhibitory peptides from diverse protein sources have been extensively studied including cereal, oilseeds, milk, and macroalgae [22]. Both PIs and associated digests were screened for their ACE inhibitory potential. Neither PICP, nor PISFE prior in vitro digestion showed ACE inhibitory activity (data not shown), but their hydrolysates exerted significant inhibitory activity, which indicates that during the in vitro digestion ACE inhibitory peptides were released from both PIs. ACE inhibition increased with increasing sample concentration resulting in a dose-dependent inhibitory response (data not shown). Values for IC_50_ were 78 µg/mL for PICP and 71 µg/mL for PISFE after pepsin digestion step, but they are increase after pancreatin digestion to values of 141 µg/mL for PICP and 92 µg/mL for PISFE (Figure 5). These reductions of inhibitory activity may be caused by a change in pH values. Similar values have also been reported for apricot seed protein isolates, where IC_50_ value after simulated gastrointestinal digestion was about 150 µg/mL [5].

## 4. Conclusions

Apricot oil cakes, obtained after both oil extraction process, cold pressing and supercritical fluid extraction contain significant amount of proteins which can be prepared by alkaline extraction followed by isoelectric precipitation method. The protein isolate possesses good functional properties and potential hypoglycemic activity. Moreover, these proteins are easily digestible. The present study will provide better knowledge about the degradation of apricot kernel protein isolate in the digestive system that would be relevant for the development of easily digestible products for human food applications. The promising bioactivity of protein hydrolysates obtained by digesting suggests their potential use as a novel nutraceutical ingredient.

## Figures and Tables

**Table 1:** Chemical compositions of apricot oil cakes and protein isolates and functional properties of protein isolates obtained from oil extraction by cold pressing (CP) process and supercritical fluid extraction (SFE).

Composition	CP	SFE
Moisture (%)	8.90 ± 0.10	6.25 ± 0.03
Crude fiber (%)	8.94 ± 0.40	8.06 ± 0.19
Crude lipids (%)	22.04 ± 0.23	6.37 ± 0.54
Total carbohydrate (%)	15.07 ± 0.01	23.23 ± 0.06
Reducing sugars (%)	6.30 ± 0.01	6.72 ± 0.02
Crude protein (%)	42.31 ± 0.53	43.43 ± 0.80
Protein content in isolates (%)	83.94 ± 0.71	70.68 ± 0.18
Amygdalin content in isolates (mg/g PI)	0.0046 ± 0.0002	<LOD
Functional properties		
Water absorption capacity (g/g PI)	0.53 ± 0.06	0.65 ± 0.033
Oil absorption capacity (g/g PI)	0.67± 0.05	0.91 ± 0.054
Foaming capacity (%)	53.33 ± 0.6	46.67 ± 0.7
Foaming stability (%)	45.33 ± 0.8	44.67 ± 0.4
Emulsion’s mean droplet diameter (µm)	3.29 ± 0.1	3.38 ± 0.4

LOD—limit of detection = 0.00024 mg/g.
